# Comparing the Efficacy of Ceftazidime and Meropenem in Treatment of Febrile Neutropenia in Pediatric Patients with Cancer 

**Published:** 2013-07-22

**Authors:** F Ferdosian, R Ghiliyan, A Hashemi, B Akhondzadeh, E Gholampoor

**Affiliations:** 1 Department of Pediatrics, Shahid Sadoughi University of Medical Science and Health Services, Yazd, Iran.; 2 Internal Medicine. Hematology oncology and Genetics Research Center Shahid Sadoughi University of Medical Sciences and Health Services, Yazd, Iran.; 3 Department of Pediatrics, Hematology, Oncology and Genetics Research Center, Shahid Sadoughi University of Medical Sciences and Health Services, Yazd, Iran.; 4 Medical Student, Shahid Sadoughi University of Medical Science and Health Services, Yazd, Iran.

**Keywords:** Ceftazidime, Meropenem, Pediatrics

## Abstract

**Background:**

In cancer patients, various infections were developed due to severe neutropenia resulted from chemotherapy. Ceftazidime is commonly used as monotherapy of cancer patients with fever and neutropenia. Meropenem is a new carbapenem with more extended antibacterial spectrum including anaerobes. It provides better coverage against gram positives. This trial compared the efficacy and safety of meropenem with ceftazidime as empirical monotherapy for febrile neutropenia in pediatric patients with cancer.

**Materials and Methods:**

A prospective, double-blind, randomized clinical trial was conducted at Departments of Pediatric Haematology/Oncology, University Hospitals, Yazd, Iran, during the years 2012 to 2013. A total of 48 cancer patients participated in the trial.

**Result:**

In this study, 26 patients (54.16%) were treated by ceftazidime and 22 patients (45.84%) by meropenem. Mean duration of fever in those who responded to treatment in ceftazidime group was 19.43+/-31.04 hours, and in meropenem group was 16.53+/-28.77 hours (P-value = 0.965).

**Conclusion:**

Finding of this study indicate that ceftazidime and meropenem have similar efficacy in treatment of fever and sever neutropenia. Due to more availability and lower cost of ceftazidime than meropenem, ceftazidime is suggested as a first line treatment in fever and neutropenia.

## Introduction

Cancer patients, whom their aggressive myelosupressive chemotherapy leads to severe neutropenia, are at high risk of developing life- threatening bacterial infections (1). Because of the defect in the inflammatory response, the classic signs of infection such as pain, heat, redness and swelling are often absent in neutropenic patients. Since fever is generally the first and almost the only sign of infection and prompt antimicrobial therapy with broad-spectrum bactericidal antibiotics is needed before the nature and susceptibility of the pathogen are known (2). Although combination antibiotic regimens have been widely used as initial therapy for febrile neutropenic cancer patients, controlled trials have demonstrated no significant differences between multidrug regimens and single agents, and monotherapy is considered a standard of treatment (3,4 and 5). Recent studies have evaluated carbapenems and third- or fourth-generation cephalosporins as initial monotherapy in febrile neutropenic cancer patients (6, 7, 8, 9). Ceftazidime , a third-generation cephalosporin, is the most commonly used agent. Its widespread use, ceftazidime, like most cephalosporin antibiotics, has only limited activity against Gram-positive bacteria, and resistance of Gram-negative organisms to ceftazidime is of concern (10). Meropenem, a new carbapenem, offers a broad spectrum of activity against both Gram-positive and Gram-negative organisms (including anaerobes) and possesses wide bactericidal activity, and drug is extremely well tolerated. Meropenem monotherapy in patients with febrile neutropenia has been evaluated in five published trials and has been shown to be as well tolerated and as effective as ceftazidime (7, 10, and 11). 

Most of these studies focused on adult patients and showed no significant advantage of any specific regiment (12, 13). We compared efficacy and safety of meropenem and ceftazidime as empirical monotherapy in pediatric patients. 

## Material and Method

This was a prospective, double-blind, randomized clinical trial that evaluated the safety and efficacy of meropenem compared to ceftazidime in the treatment of febrile episodes in neutropenic patients. This study included febrile and neutropenic patients (< 14 years old) who had been treated with conventional or high-dose chemotherapy for hematological and non hematological malignancy at the Departments of Pediatric Hematology/Oncology, University Hospital, and Yazd, Iran during the years 2012 to 2013. Patients were eligible for entry into the study if they had fever defined as an elevation to 38.5°Cover at least a 4 h period or a single temperature elevation above 39°C, and with neutropenia defined as an absolute neutrophil count (ANC) of <0.5 _ 109/L at admission or >0.5 _ 109/L expected to fall within the next 24–48 h to <0.5 _ 109/L and with a presumptive infection (i.e. exclusion of febrile episodes likely due to neoplastic disease or to drug or blood product administration). Patients who had repeated febrile neutropenic episodes during consecutive cytostatic treatment periods could be entered more than once. Patients were excluded from this study if they had received any intravenous (iv) or oral antibiotic medication other than antimicrobial prophylaxis during the 48 h preceding admission, if they had a history of sensitivity to penicillin, cephalosporin, or carbapenem antibiotics; marked hepatic disease, infectious hepatitis, or renal failure ,or positive human immunodeficiency virus status; CNS disease, including a history of seizures or any condition that increased the risk of seizures; chronic lymphocytic leukemia; cystic fibrosis, and if they were using immunomodulators. [World Health Organization (WHO) toxicity scale > 3] (14).

After obtaing a detailed history, eligible patients underwent a physical examination. Clinical evaluation of signs and symptoms, culture and susceptibility testing, chest Xray

(LRTI as clinically indicated), Clinical laboratory tests, including hematologic (hemoglobin, hematocrit, WBC count, total and differential, platelet count, prothrombin time) and serum chemistry tests (AST, ALT, total bilirubin, alkaline phosphatase, BUN, creatinine) as well as urinalysis were performed during treatment and at the end of therapy.

All possible sources of infection were investigated. During treatment, the following procedures were performed: temperature, physical examination, clinical evaluation of signs and symptoms, culture and susceptibility testing, chest X-ray (LRTI), laboratory tests including hematology and serum chemistry and urinalysis. Any clinical adverse events occurring in association with test drug administration, whether believed by the investigator to be related or unrelated to the test drug(s) were recorded.

After completing a pretherapy evaluation, patients were allocated at random for each neutropenic episode to receive an intravenous infusion of either meropenem [60 mg/kg/day in three single doses (SDs), SD < 1.0 g) or ceftazidime (150 mg/kg/day in 3 SDs, SD < 2.0 g). They were administered as initial monotherapy of the sequential regimen. Antibiotics were administered intravenously to all patients for at least 24 h after the cessation of fever and for a minimum of 72 h. Blinded drug was distributed based on a schedule that provided a stratified, balanced and block random assignment. As patients were enrolled for specific neutropenic episodes, they were assigned for the next available number and associated randomized treatment. Once assigned to receive meropenem or ceftazidime for a specific episode, patients continued to receive regimen until the febrile episode resolved or treatment was discontinued or modified. No additional antibiotic therapy was permitted with administration of meropenem or ceftazidime. When any new antibiotic was added, patient episodes were classified as failures. Treatment with either drug could be discontinued because of an intolerable side effect, disease exacerbation, or when patients elected to withdraw from therapy.

We evaluated and compared duration of fever and neutropenia after initial therapy, recurrence of infection, duration of admission, response to treatment (amelioration of fever after 72 hours) and side effects of drugs in both groups. 

**Statistical analysis**

Safety analysis was performed for the intent-to-treat population (all randomized patients who received trial medication). All analyses were performed using a two-tailed Student’s *t *test with an alpha level of 0.05. 

## Results

Forty eight patients with neutropenia (26 scheduled to receive meropenem and 22 scheduled to receive ceftazidime) were enrolled in the trial. The treatment groups formed by randomization of patient episodes were well-balanced with respect to sex and age (Table I). Mean of age of meropenem group was 5.59+/-2.87 and in ceftazidime group was 6.12+/-1.94. There was no significant difference between 2 groups (P-value= 0.470). 

There was hematological malignancy in 19(86.4%) of meropenem group and 23(92%) of ceftazidime group that there was no significant difference between 2 groups (P-value = 0.654).

Duration of fever in meropenem ceftazidime groups were 28.77+/-16.53 hours and 31.04+/-19.43 hours respectively. There was no significant difference between 2 groups (P-value = 0.965).

There was recurrence of infection 9.1% of meropenem group and 15.4% of ceftazidime group that there was no significant difference between 2 groups (P-value = 0.674).

Duration of neutropenia after initial therapy, in meropenem group was 3.91+/-2.31 days and in ceftazidime group was 3.92+/-1.69 days. There was no significant difference between 2 groups (P-value = 0.735).

 Duration of admission in meropenem group was 6.86+/-4.19 days and in ceftazidime group was 7.19+/-4.08 days. There was no significant difference between 2 groups (P-value = 0.624).

Two (7.7%) patients in ceftazidime group and one (4.5%) patients in meropenem group did not response to treatment. There was no significant difference between 2 groups (P-value = 0.81) ( Table.II).

Only one case with mild diarrhea recorded in ceftazidime group that did not lead to withdrawal from therapy. Another group didn’t show any side effect. There was no significant difference between 2 groups (P-value = 0.795). 

**Table I:** Demographic and pretreatment characteristics for patient episodes

**characteristics**	**Patients**			
	** Meropenem **	**N=22**	**Ceftizidine **	**N=26 **
	**No**	**%**	**No**	**%**
**Sex**				
**Male** **female**	13 9	29.16 40.9	18 8	69.26 30.8
**Age(years)**				
**Mean** **SD**		5.59 2.87		6.12 1.94
**Underlying disease**				
**Leukemia** **Lymphoma** **Hepatoblastoma** **Neuroblastoma** **Ewding sarcoma**	16 3 1 1 1	72.7 13.6 4.5 4.5 4.5	21 3 1 1 0	80.7 11.5 3.8 3.8 -

**Table II:** Comparing the effects of drugs in two groups

	**ceftazidime**		**meropenem**		**P-value**
	**mean**	**SD**	**mean**	**SD**	
**Duration of fever**	31/04	19/43	28/77	16/53	0/965
**Duration of admission**	7/19	4/08	6/86	4/19	0/624
**Duration of neutropenia**	3/92	1/69	3/91	2/31	0/735

## Discussion

Several studies have evaluated the use of broad-spectrum antibiotics such as third- or fourth-generation cephalosporins or carbapenems as empirical monotherapy for febrile neutropenic cancer patients. The reported success rates range from 48 to 82% for meropenem or imipenem (6,7,15,16,17,18,19) and from 38 to 66% for ceftazidime or cefepime (3,7,9,17,18,20). This study compared the safety and efficacy of ceftazidime and meropenem, as empiric therapy for the treatment of high-risk febrile neutropenic patients. By using a double-blind trial design, we reduced the likelihood that inappropriate treatment modifications by the investigators would bias the results (21).

Because definitions of response are not consistent among published trials, it is difficult to directly compare results from this trial with other trials testing the value of empirical antibiotic therapy for febrile neutropenia but despite theoretical considerations (6, 11), we observed no advantage of meropenem over ceftazidime as empirical therapy in these patients. Despite our results, studies of Feld and Hung KC and coworkers showed, Meropenem may be more effective than ceftazidime specially in two subgroups of high-risk patients, (sever neutropenia and bone marrow transplantation) (10, 22). Other similar study in pediatric sections or wards showed, the success rate of the initial monotherapy differed significantly between the two drugs and was 55.8% in the meropenem and 40.0% in the ceftazidime group (23). This difference may be due to small number of cases respect to age (adults, children or both), underlying malignant disease (hamatological, non-hamatological malignancies or both), disease stage (newly diagnosed cancer patients, relapsed patients or both) and intensity of chemotherapy (less myelotoxic or myeloablative therapy associated with a difference non-hamatological toxicity).

Similar to our study K.Serefhanoghlu and coworkers (24) showed, duration of fever and neutropenia after initial therapy, and antimicrobial therapy did not show difference in both group, but Fleschhak and coworkers significantly were observed longer duration of fever and antimicrobial therapy in the ceftazidime arm than in the meropenem arm(median 5 versus 4 days, and 7 versus 6 days). In later or furture study most isolated pathogens were Gram-positive organisms, and efficacy of meropenem was better, but in our study pathogens were not recognized.

 Nausea, vomiting, abdominal pain, headache, rash and vertigo were established side effects of therapy with both drugs, but they were well tolerated (25). In our study and all of other trials the observed toxicity was low in both drug groups and did not lead to withdrawal from therapy (9,10,19,25,26). 

## Conclusion

In conclusion, empirical monotherapy with meropenem or ceftazidime are effective and well tolerated for the treatment of febrile neutropenic episodes in paediatric cancer patients. On the other hand meropenem is more useful as empirical monotherapy in febrile pediatric cancer patients with severe neutropenia and bonemarrow transplantation, thus it is better to use meropenem in two subgroups of high- risk patients to decrease drug resistance. 
